# One-year intradialytic leg exercises with resistance bands and fat mass increase in elderly hemodialysis patients: a retrospective study

**DOI:** 10.1186/s41100-021-00341-z

**Published:** 2021-05-05

**Authors:** Masahiro Kato, Masanori Shibata, Kazuaki Asai, Kumi Harada, Isao Ito, Hisae Tawada, Kojiro Nagai, Shinkichi Taniguchi

**Affiliations:** 1 Department of Hemodialysis, Koujukai Rehabilitation Hospital, 85 Kouden, Kunotsubo, Kita-Nagoya, Aichi 481-0041 Japan; 2 Department of Nephrology, Institute of Biomedical Sciences, Tokushima University Graduate School, 3-18-15, Kuramoto-cho, Tokushima, 770-8503 Japan; 3 Department of Dermatology, Wakayama Medical University, Wakayama, Japan

**Keywords:** Resistance exercise, Dialysis, Nutrition, Multi-frequency bioimpedance, Body composition

## Abstract

**Background:**

Intradialytic exercises are recommended to be available as a treatment for enhancing physical functioning. However, there have been few reports which evaluated the results of long-term mild intradialytic exercises in elderly patients. The purpose of this study is to investigate the changes in body weight, body composition, and laboratory data in elderly hemodialysis patients after 1-year intradialytic leg exercises with resistance bands.

**Methods:**

A retrospective study. Twenty-one outpatients, aged 65 or older (mean ± SD, 75.2 ± 5.1 years), received intradialytic leg exercises with resistance bands for a year were analyzed. The values of dry weight, body composition, and laboratory data were collected from the year-ago period, at baseline and 1 year after baseline. Fat and muscle mass were evaluated by using a multi-frequency bioimpedance device.

**Results:**

Physical performance changed and body weight increased after 1-year resistance band exercises. However, the participants gained fat mass, not muscle mass. Although the changes in biochemical data related to protein intake were equivocal, triglyceride levels increased significantly after 1-year exercises. An elevation in serum creatinine levels was observed, even if solute clearance increased significantly.

**Conclusions:**

One-year intradialytic leg exercises with resistance bands may have a potential clinical benefit for body mass index even in elderly hemodialysis patients. However, optimal dietary modification is needed to achieve a balanced increase of muscle and fat mass. An increase of serum creatinine levels does not always mean muscle mass hypertrophy.

**Supplementary Information:**

The online version contains supplementary material available at 10.1186/s41100-021-00341-z.

## Background

The use of intradialytic exercises, as a novel and efficient use of time during hemodialysis, is well established in Australia and some European nations [1]. The Renal Association in the UK provided clinical practice guidelines on hemodialysis and recommended exercise delivered during hemodialysis. Intradialytic exercises improved muscle strength and functional capacity especially in young patients [2–5]. On the other hand, in elderly patients, even if exercise was combined with oral nutritional support for several months, it was difficult to improve physical performance and increase muscle mass [6, 7]. However, so far, there have been few reports which investigated the results of 1-year continuous intradialytic exercises only for legs about the changes in body weight and body composition in elderly patients excluding seasonal influences [8].

Multi-frequency bioimpedance analysis is known to be useful for assessing volume status using the values of total body water (TBW), intracellular water (ICW), and extracellular water (ECW) in hemodialysis patients [9]. It also provides the information of body composition such as fat mass in hemodialysis patients [10]. The fat tissue index (FTI) and skeletal muscle mass index (SMI) have been used to evaluate fat mass and muscle mass, respectively, and the SMI is used for the diagnosis of sarcopenia when muscle mass is measured by bioelectrical impedance analysis [11–14].

In this study, we investigated the changes in body weight, body composition, and laboratory data in elderly hemodialysis patients to know whether long-term intradialytic exercises may have some benefits for the entire body, even if the exercises were only for legs.

## Methods

### Design and participants

**Fig. 1:**
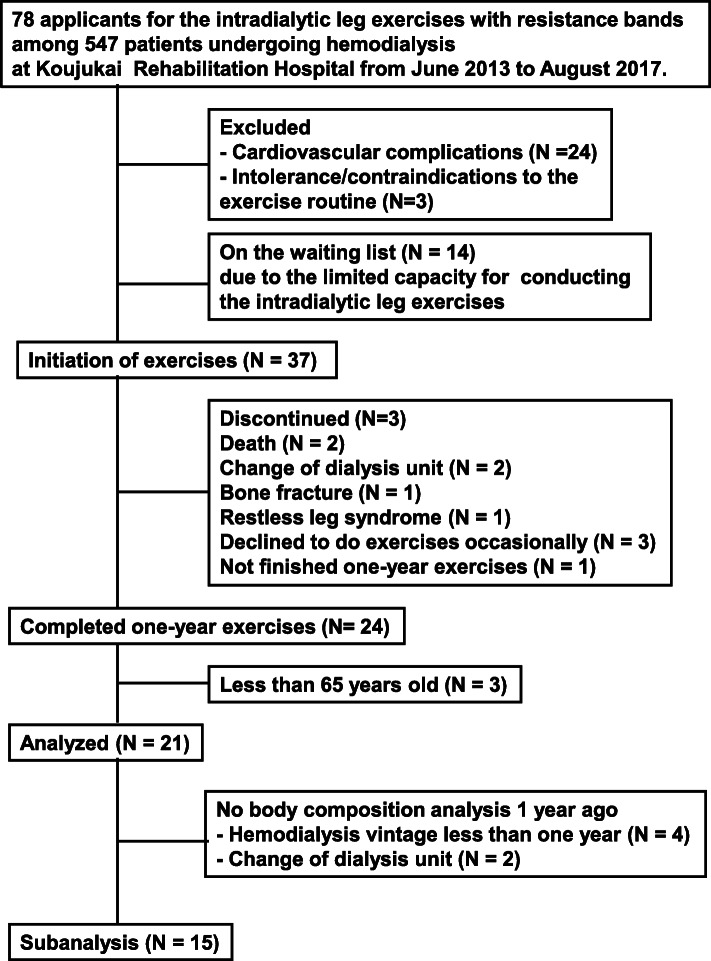
A flow diagram of the patient selection and exclusion process

**Fig. 2:**
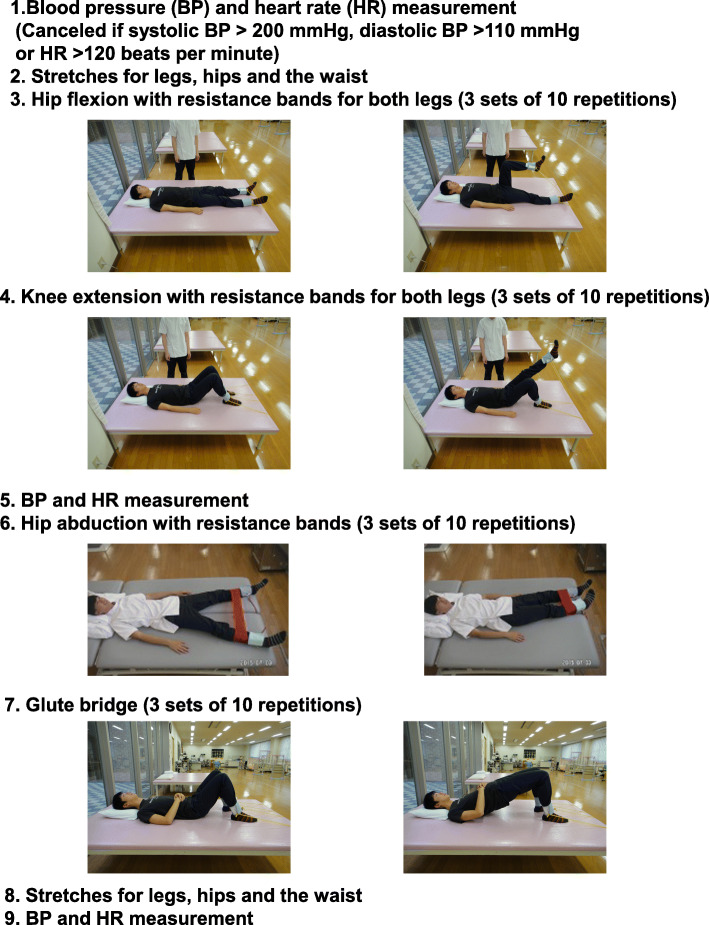
Prescriptions for intradialytic leg exercises with resistance bands

This is a retrospective study. A flow diagram of the patient selection and exclusion process are shown in Fig. 1. From June 2013 to August 2017, there were 547 patients undergoing hemodialysis at Koujukai Rehabilitation Hospital. We explained the contents of intradialytic leg exercises with resistance bands by using the brochure of the prescriptions for the exercises (Fig. 2). Briefly, after stretches for legs, hip, and waist, four kinds of intradialytic exercises were performed under the guidance of physical therapists. The target resistance strength was set to rating 11 to 13 (light to fairy hard) based on the Borg rating of perceived exertion [15]. The exercises were stopped if they had symptoms, signs related to cardiovascular disease, or extremely high or low blood pressure according to the statement from the American Heart Association [16]. It took around 40 min within the first half of each hemodialysis treatment. Seventy-eight applicants were screened by the doctors to conduct the exercises safely. Twenty-seven patients were excluded mainly due to cardiovascular complications such as ischemic heart disease and severe hypertension. Fourteen were still on the waiting list, because we set up one physical therapist for three patients to provide the appropriate exercises. Thirty-seven could initiate the intradialytic exercises. However, 13 could not finish 1-year exercises due to death or intolerance to continue exercises. Finally, there were 24 outpatients who completed 1-year exercises. Among them, this analysis includes 21 outpatients undergoing hemodialysis aged 65 or older. Every participant received 4-h hemodialysis that started at the same time, three times a week.

A subanalysis was also done using 15 outpatients who had underwent hemodialysis at Koujukai Rehabilitation Hospital for a year when they started the exercises (hereafter, baseline), because they could receive body composition analysis 1 year before baseline. Four were excluded because their dialysis vintages were less than 1 year. Two were excluded because they moved from the other dialysis unit within 1 year before baseline.

In order to support the adequacy of the exercises, we performed 10-m walk test (10MWT), timed up and go test (TUG) [17], and measured percent knee extension muscle power to dry body weight (weight bearing index, WBI) [18] at baseline, 3 months, 6 months, and 1 year after baseline. These tests were examined on non-dialysis day. WBI was examined three times and the mean value was collected as the muscle strength of lower extremities using a hand-held dynamometer (μTas F-1, Anima, Inc. Tokyo, Japan).

Demographic characteristics were collected at baseline. Blood samples for laboratory data were obtained from arteriovenous shunt just before starting the first hemodialysis session of the week. Normalized protein catabolic rate (nPCR) and creatinine generation rate (CGR) were calculated using the method of Shinzato [19, 20]. Dry weight was determined by considering cardiothoracic ratio, blood pressure, clinical symptoms, and physical findings such as edema. Body composition was measured by using a multi-frequency bioimpedance device, MLT-50 (Sekisui Medical Co., Ltd. Tokyo, Japan.) immediately following the last hemodialysis session of the week [21]. Skeletal muscle mass was estimated using the following formula: skeletal muscle mass (kg) = 9.52 + 0.331 × ICW (L) + 2.77 (if male) + 0.180 × post-dialysis weight (kg) − 0.133 × age (years) [22]. Then, FTI and SMI, which represent respective tissue masses adjusted for height squared, were calculated. Erythropoietin usage was adjusted by using a dose conversion ratio of 40 and 60 μg of darbepoetin alpha: 9000 and 12,000 international units of epoetin alfa according to the manufacturer’s instruction.

### Statistical analysis

All values are expressed as mean ± SD. Statistical analysis was performed using SPSS for Windows version 13.0 (SPSS, Inc., Chicago, IL, USA). The yearly changes were analyzed using paired *t* test or Wilcoxon signed-ranks test, as appropriate. Significance was defined by *P* less than 0.05.

## Results

### Characteristics of study participants at baseline

They were aged 65 to 82 years (mean ± SD, 75.2 ± 5.1 years), including 15 males and 6 females. Dialysis vintage ranged from 1 to 200 months (48.3 ± 52.8 months). Body mass index (BMI) varied from 15.4 to 29.7 kg/m^2^ (20.9 ± 3.1 kg/m^2^). Eight participants suffered from diabetic nephropathy (Table 1).

**Table 1:** Demographic and clinical characteristics of study participants at baseline

Patients, *n*	21
Age, years	75.2 ± 5.1
Female, *n* (%)	6 (28.6%)
Diabetic nephropathy, *n* (%)	8 (38.1%)
Hemodialysis vintage, months	48.3 ± 52.8
Height, cm	159.4 ± 9.0
Dry weight, kg	53.1 ± 8.9
Body mass index, kg/m^2^	20.9 ± 3.1
Kt/V	1.43 ± 0.24
nPCR, g/kg/day	0.87 ± 0.16
Creatinine generation rate, %	105.2 ± 26.2
Hemoglobin, g/dL	10.5 ± 0.7
Albumin, g/dL	3.51 ± 0.32
Urea nitrogen, mg/dL	64.4 ± 13.8
Creatinine, mg/dL	9.2 ± 1.9
Phosphate, mg/dL	4.60 ± 0.91
Total cholesterol, mg/dL	150.7 ± 27.4
LDL cholesterol, mg/dL	83.6 ± 21.8
HDL cholesterol, mg/dL	48.1 ± 12.0
Triglyceride, mg/dL	100.9 ± 46.0
C-reactive protein, mg/dL	0.11 ± 0.12
FTI, kg/m^2^	5.49 ± 2.82
SMI, kg/m^2^	6.48 ± 0.85
ECW/TBW ratio, %	35.9 ± 6.2

*nPCR* normalized protein catabolic rate, *FTI* fat tissue index, *SMI* skeletal muscle mass index, *ECW/TBW* extracellular water/total body water

All values are expressed as mean ± SD

### Physical performance of participants after 1-year intradialytic resistance exercises

**Fig. 3:**
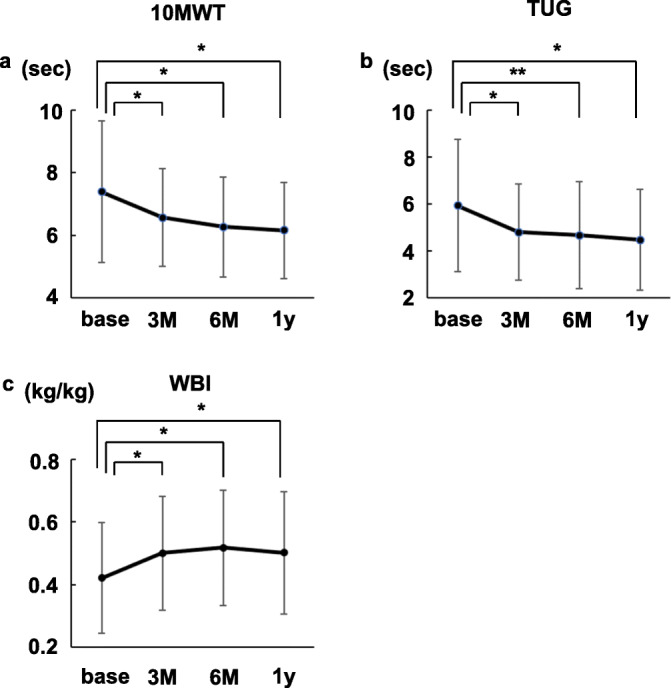
Physical performance in participants undergoing resistance exercises. **a** 10-m walk test (10MWT) results. **b** Timed up and go test (TUG) results. *N* = 21 at base and 1y. *N* = 20 at 3 and 6 M, because we forgot to perform the tests for one participant. **c** Weight-bearing index (WBI). *N* = 21 at base, 3M, and 1y. *N* = 20 at 6M because we failed to remember to measure the value for one participant. base, at the time of the exercise initiation. 3M, 6M, and 1y, 3, 6 months, and 1 year after the exercise initiation. *P<0.01. **P<0.05

In order to support the adequacy of intradialytic leg exercises, 10MWT and TUG were performed and WBI was measured. All of the test results changed significantly throughout 1 year (Fig. 3).

### Body weight and fat mass increased in participants undergoing resistance exercises

**Fig. 4:**
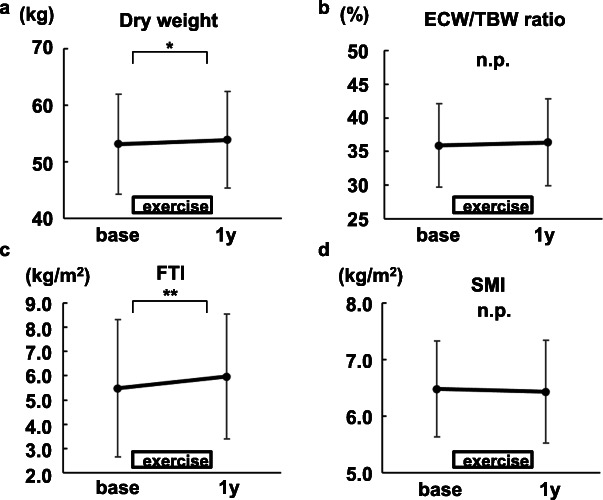
The changes in dry weight and body composition by intradialytic leg exercises with resistance bands (*N*=21). **a** Dry weight. **b** Extracellular water/total body water (ECW/TBW) ratio. **c** Fat tissue index (FTI). **d** Skeletal muscle mass index (SMI). Mean ± SD are shown. base, at the time of the exercise initiation. 1y, 1 year after the exercise initiation. *P<0.01. **P<0.05

Dry weight increased significantly after 1-year exercises (Fig. 4a). It was not due to volume overload based on the ECW/TBW ratio, which is one of the markers to assess dry weight (Fig. 4b) [23]. As shown in Fig. 4c, d, FTI increased significantly after 1-year exercises, while SMI did not.

### The changes in laboratory data related to nutrition in participants undergoing resistance exercises

**Fig. 5:**
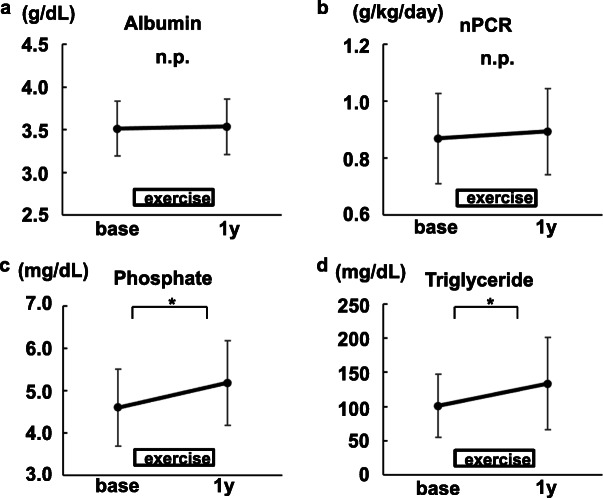
The changes in biochemical data related to dietary intake (*N*=21). **a** Albumin. **b** Normalized protein catabolic ratio (nPCR). **c** Phosphate. **d** Triglyceride. Mean ± SD are shown. base, at the time of the exercise initiation. 1 y, 1 year after the exercise initiation. *P<0.01

The values of albumin and nPCR did not increase after 1-year exercises (Fig. 5a, b). Phosphate levels increased significantly (Fig. 5c). For your information, six participants took more phosphate binders, two took fewer binders and the other 13 participants used the same dose of the drugs 1 year after baseline (Additional file 1). Triglyceride levels also increased significantly (Fig. 5d).

### The changes of serum creatinine levels

**Fig. 6:**
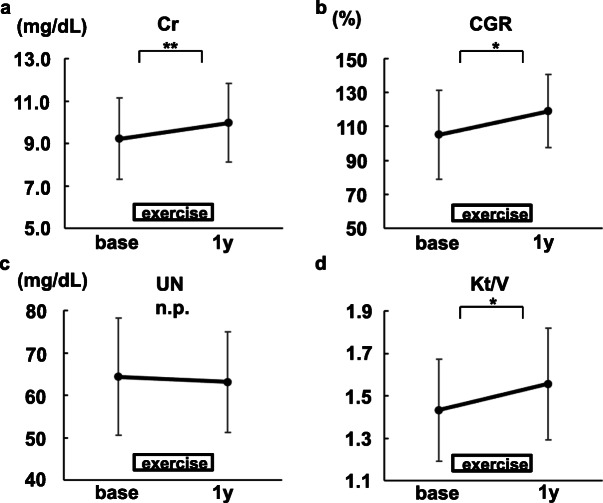
The changes in biochemical data related to serum creatinine levels (*N*=21). **a** Creatinine. **b** Creatinine generation ratio (CGR). **c** Urea nitrogen (UN). **d** Kt/V. Mean ± SD are shown. base, at the time of the exercise initiation. 1y, 1 year after the exercise initiation. *P<0.01. **P<0.05

One year after baseline, higher serum creatinine levels were observed in accordance with an increase of creatinine generation rate (Fig. 6a, b). Elevated serum creatinine levels were not because of inadequate solute clearance (Fig. 6c, d).

### The management of anemia

We investigated the anemia control after 1-year exercises. The levels of hemoglobin did not change. Regarding erythropoietin treatment, seven patients needed larger dose, six patients took smaller dose, and the rest of eight patients took the same dose of erythropoietin administration (Additional file 1).

### Confirmation of the changes after long-term mild resistance exercises

**Fig. 7:**
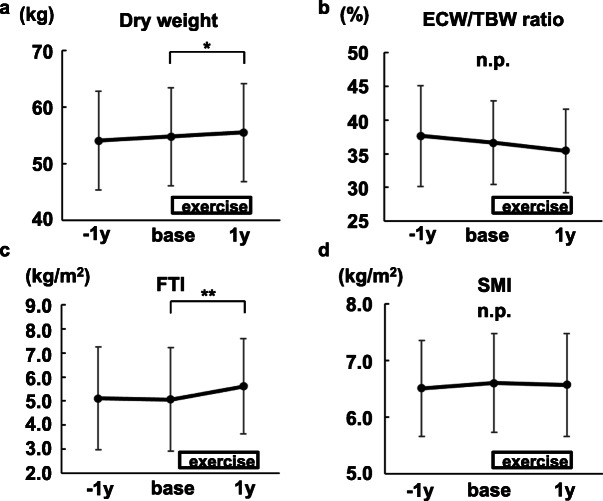
The changes in dry weight and body composition before and after intradialytic leg exercises with resistance bands (*N*=15). **a** Dry weight. **b** Extracellular water/total body water (ECW/TBW) ratio. **c** Fat tissue index (FTI). **d** Skeletal muscle mass index (SMI). Mean ± SD are shown. −1y, 1 year before the exercise initiation. base, at the time of the exercise initiation. 1y, 1 year after the exercise initiation. *P<0.01. **P<0.05

**Fig. 8:**
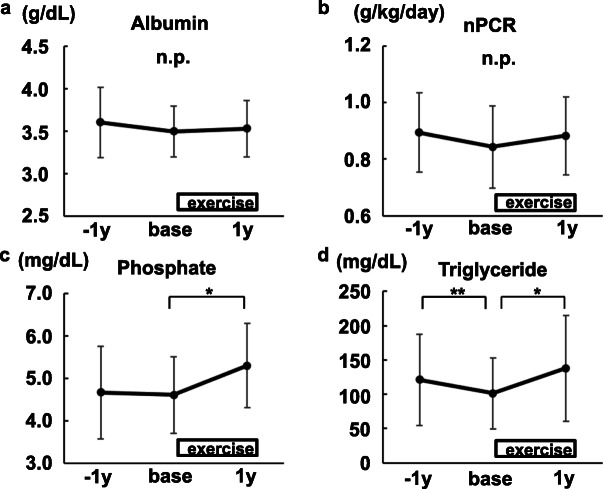
The changes in biochemical data related to dietary intake (*N*=15). **a** Albumin. **b** Normalized protein catabolic ratio (nPCR). **c** Phosphate. **d** Triglyceride. Mean ± SD are shown. −1y, 1 year before the exercise initiation. base, at the time of the exercise initiation. 1y, 1 year after the exercise initiation. *P<0.01. **P<0.05

**Fig. 9:**
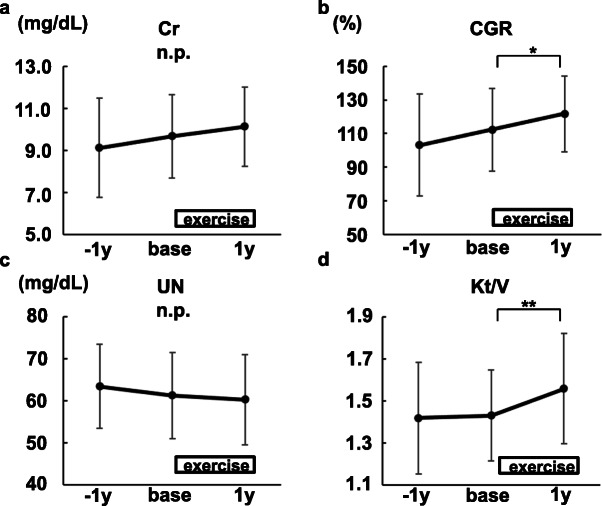
The changes in biochemical data related to serum creatinine levels (*N*=15). **a** Creatinine. **b** Creatinine generation ratio (CGR). **c** Urea nitrogen (UN). **d** Kt/V. Mean ± SD are shown. −1y, 1 year before the exercise initiation. base, at the time of the exercise initiation. 1y, 1 year after the exercise initiation. *P<0.01. **P<0.05 Fig. 9

A subanalysis was done using 15 participants whose data 1 year before baseline were available. It confirmed that the changes of dry weight, fat mass, and nutritional status were not natural course in the elderly hemodialysis patients undergoing 1-year mild continuous exercises only for legs (Figs. 7, 8, and 9, Table 2).

**Table 2:** Demographic and clinical characteristics of study participants at baseline in a subanalysis

Patients, *n*	15
Age, years	76.1 ± 4.2
Female, *n* (%)	2 (13.3%)
Diabetic nephropathy, *n* (%)	5 (33.3%)
Hemodialysis vintage, months	61.7 ± 57.1
Height, cm	162.0 ± 8.3
Dry weight, kg	54.8 ± 8.7
Body mass index, kg/m^2^	20.8 ± 2.6
Kt/V	1.43 ± 0.22
nPCR, g/kg/day	0.84 ± 0.15
Creatinine generation rate, %	112.4 ± 24.6
Hemoglobin, g/dL	10.5 ± 0.8
Albumin, g/dL	3.49 ± 0.30
Urea nitrogen, mg/dL	61.3 ± 10.3
Creatinine, mg/dL	9.7 ± 2.0
Phosphate, mg/dL	4.61 ± 0.91
Total cholesterol, mg/dL	148.7 ± 30.6
LDL cholesterol, mg/dL	83.7 ± 25.2
HDL cholesterol, mg/dL	45.9 ± 10.3
Triglyceride, mg/dL	101.3 ± 51.5
C-reactive protein, mg/dL	0.12 ± 0.12
FTI, kg/m^2^	5.07 ± 2.16
SMI, kg/m^2^	6.60 ± 0.87
ECW/TBW ratio, %	36.6 ± 6.2

*nPCR* normalized protein catabolic rate, *FTI* fat tissue index, *SMI* skeletal muscle mass index, *ECW/TBW* extracellular water/total body water

All values are expressed as mean ± SD

## Discussion

In this study, we could find an increase of dry weight and fat mass, not muscle mass after long-term intradialytic leg exercises with resistance bands in elderly hemodialysis patients. It was probably due to the participants’ dietary intake.

The most important finding is that, even in elderly participants, long-term intradialytic resistance exercises only for legs may have a benefit for body mass index (BMI), one of the important factors related to mortality in hemodialysis patients [24]. However, not every exercise always is involved in muscle hypertrophy. Our data suggested that the participants had carbohydrate and fat rich diets instead of protein rich ones. All of the participants received the nutrition education for hemodialysis patients. The education emphasized the importance of controlling potassium and phosphate levels, which could affect their dietary habits. However, in order to control sarcopenia, it is important to design a balanced diet or oral supplement, and prescribe optimal phosphate binders to take advantage of the exercises and obtain muscle hypertrophy without causing hyperphosphatemia [25].

In general, exercise training is aimed to improve physical performance and to decrease body weight and fat mass even in the elderly [26, 27]. However, exercise training is suggested to increase dietary intake, body weight, and fat mass under certain conditions. Vitale et al. showed that home-based resistance training for older subjects (66 ± 4 years) during home confinement due to the COVID-19 outbreak in Italy increased total body fat significantly, partially because a reduced daily physical activity regimen and altered diet pattern [28]. Yamamoto et al. reported that total calorie and protein intakes were significantly higher after the preoperative exercise and nutritional support program (median duration: 16 days) for elderly sarcopenic patients with gastric cancer aged 65 years or older [29]. In hemodialysis patients, Hristea et al. investigated the effects of a 6-month intradialytic cycling program combined to nutritional support on protein energy wasting, physical functioning, and quality of life in older hemodialysis patients (69.7 ± 14.2 years) by a randomized design. A higher total energy intake was shown in the group undergoing exercise with nutritional support, compared to the intake in the group with nutritional support only. FTI increased, but not significantly in the group undergoing exercise with nutritional support [30]. Moreover, Dong et al. demonstrated the effects of intradialytic resistance exercise on biochemical data and body composition in maintenance hemodialysis patients (median, 60 years) with sarcopenia by a randomized controlled trial. Twelve-week intradialytic resistance exercise increased Kt/V and serum albumin in exercise group compared to those in control group significantly. It also increased BMI and FTI in exercise group significantly [31]. Johansen et al. also reported the effects of 3-month intradialytic resistance exercise of the lower extremities in patients undergoing hemodialysis (control group, 56.8 ± 13.8 years; exercise group, 54.4 ± 13.6 years). The exercise resulted in a significant increase in body fat mass [32]. These previous reports can support the results obtained in this study.

Of note, serum creatinine levels also increased after the exercises, even if Kt/V became higher. It is natural because creatinine is generated from muscles by the exercises. However, we sometimes misunderstand that serum creatinine levels are proportional to muscle mass. Muscle mass is one of the important factors to explain serum creatinine levels. However, we must realize the exercises can increase serum creatinine levels by increasing muscle breakdown [33].

Resistance training can increase hemoglobin [34, 35]. However, we could not find the preferable change in the management of anemia in this study. It is probably because our resistance exercises were relatively mild and hemodialysis patients have many factors influencing hemoglobin levels such as blood loss and nutrition intake.

The limitations of this study should be addressed. First, this study included only a small number of participants. Second, we did not measure hand grip strength. We need further studies to know the effect of long-term intradialytic exercises on sarcopenia. Third, we do not have detailed diet histories of the participants. Therefore, we assumed the dietary contents from laboratory data.

## Conclusions

Body weight and fat mass increased even in elderly hemodialysis patients undergoing long-term intradialytic leg exercises with resistance bands. It is important to control the dietary contents to take advantage of the exercises and gain balanced body composition to fight sarcopenia. Caution will be needed when we interpret the meaning of serum creatinine levels.

## Supplementary Information

**Additional file 1.** Subanalysis of 21 and 15 patients.

## Data Availability

All the datasets shown in this manuscript are available in Additional file 1.

## Abbreviations

10MWT: 10-m walk test
TUG: Timed up and go test
WBI: Weight bearing index
TBW: Total body water
ICW: Intracellular water
ECW: Extracellular water
FTI: Fat tissue index
SMI: Skeletal muscle mass index
nPCR: Normalized protein catabolic rate
CGR: Creatinine generation rate
BMI: Body mass index
